# DHX36 maintains genomic integrity by unwinding G‐quadruplexes

**DOI:** 10.1111/gtc.13061

**Published:** 2023-08-26

**Authors:** Ayaka Mizumoto, Yuta Yokoyama, Tomoichiro Miyoshi, Masahiro Takikawa, Fuyuki Ishikawa, Mahito Sadaie

**Affiliations:** ^1^ Department of Gene Mechanisms, Graduate School of Biostudies Kyoto University Kyoto Japan; ^2^ Department of Therapeutic Oncology, Graduate School of Medicine Kyoto University Kyoto Japan; ^3^ Department of Applied Biological Science, Faculty of Science and Technology Tokyo University of Science, Noda Chiba Japan; ^4^ Department of Stress Response, Radiation Biology Center, Graduate School of Biostudies Kyoto University Kyoto Japan; ^5^ Laboratory for Retrotransposon Dynamics RIKEN Center for Integrative Medical Sciences Yokohama Japan

**Keywords:** cell growth, DHX36, DNA damage, DNA helicase, G‐quadruplex

## Abstract

The guanine‐rich stretch of single‐stranded DNA (ssDNA) forms a G‐quadruplex (G4) in a fraction of genic and intergenic chromosomal regions. The probability of G4 formation increases during events causing ssDNA generation, such as transcription and replication. In turn, G4 abrogates these events, leading to DNA damage. DHX36 unwinds G4‐DNA in vitro and in human cells. However, its spatial correlation with G4‐DNA in vivo and its role in genome maintenance remain unclear. Here, we demonstrate a connection between DHX36 and G4‐DNA and its implications for genomic integrity. The nuclear localization of DHX36 overlapped with that of G4‐DNA, RNA polymerase II, and a splicing‐related factor. Depletion of DHX36 resulted in accumulated DNA damage, slower cell growth, and enhanced cell growth inhibition upon treatment with a G4‐stabilizing compound; DHX36 expression reversed these defects. In contrast, the reversal upon expression of DHX36 mutants that could not bind G4 was imperfect. Thus, DHX36 may suppress DNA damage by promoting the clearance of G4‐DNA for cell growth and survival. Our findings deepen the understanding of G4 resolution in the maintenance of genomic integrity.

## Graphical abstract

This research explored the in vivo functions of DHX36 and its relationship with G4‐DNA, uncovering a significant connection between DHX36 and G4‐DNA and its importance for maintaining genomic stability.

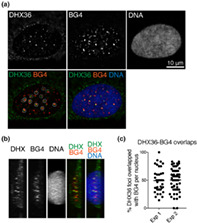

## 1 INTRODUCTION

Guanine‐rich stretches of ssDNA or RNA can potentially form a G‐quadruplex (G4), which is a layered structure consisting of at least three G‐quartets, having four guanine bases connected by Hoogsteen bonds (Bochman et al., 2012). Various G4 topologies can exist depending on the strand direction and spacer length between the guanines and whether they are formed by intra‐ or inter‐molecular association. G4 is possibly self‐assembled on the oligonucleotides containing putative G‐quadruplex–forming sequences (PQS; G _≥ 3_ N _x_ G _≥ 3_ N _x_ G _≥ 3_ N _x_ G _≥ 3_) under physiological buffer conditions in the presence of cations (Bochman et al., 2012). Owing to its high thermodynamic stability, assembled G4 needs to be resolved enzymatically. In vitro methods have been developed for monitoring the formation of G4 (Balasubramanian et al., 2011; Bryan & Baumann, 2011). Enzymatic activities that resolve G4 have been demonstrated using these methods. These enzymes include DNA helicases with G4‐binding and unwinding activities, such as BLM, WRN, PIF1, FANCJ, XPD, DNA2, and RTEL1 (Bochman et al., 2012; Maizels, 2015).

In vivo G4 formation is predicted using in silico analyses or by fluorescence imaging, immunoprecipitation, or pull‐down experiments using valuable tools—such as immunoglobulins and single‐chain variable fragments (scFv) that specifically recognize G4 (Henderson et al., 2013), G4‐binding chemical compounds (Mendoza et al., 2016), or G4‐binding proteins (Maizels, 2015). Using these tools, G4 sites are identified by immunoprecipitation or pull‐down against purified genomic DNA or chromatin, and a significant fraction of these sites recapitulate the PQS (Chambers et al., 2015; Hänsel‐Hertsch et al., 2016; Lam et al., 2013; Muller et al., 2010). PQS are overrepresented in the regulatory regions of genes (e.g., promoters, introns, or untranslated regions [UTRs]), including oncogenes, repetitive regions (e.g., telomeres and rDNA), and replication origins (Maizels & Gray, 2013). Genome‐wide G4 mapping in human cells using antibodies revealed the presence of G4s in gene regulatory regions and telomeres (Hänsel‐Hertsch et al., 2016; Liu et al., 2016). Many G4 are mapped around transcription start sites, and the frequency of G4 formation positively correlates with transcriptional levels of the corresponding genes (Spiegel et al., 2021; Zheng et al., 2020). Fluorescence labeling of G4‐DNA using antibodies shows granule‐like signals in the nuclei or on chromosomes; some signals are located on telomeres or centromeres (Biffi et al., 2013; Henderson et al., 2013). Visualization of G4‐DNA using fluorescently‐labeled compounds shows larger signals located in the nucleoli, along with some smaller signals in the nuclei (Rodriguez et al., 2012), or uniformly distributed signals throughout the nuclei (Shivalingam et al., 2015). However, the subcellular or genomic locations of many uncharacterized signals obtained using in vivo imaging are poorly understood.

Accumulating evidence shows that G4 formed in or around gene bodies regulates gene activity by promoting or suppressing transcription (Bochman et al., 2012; Mendoza et al., 2016). Despite these biological implications, G4 sterically hinders DNA replication and transcription (Bochman et al., 2012; Maizels, 2015). Stalling of these biological events increases the risk of genotoxic damages; inadequate clearance of G4 structures possibly results in accumulation of such damages. Moreover, ssDNA generation by the machinery involved in these events can result in G4 formation. Supporting the notion that G4 is stable and enzymatic activity is required for its clearance, deficiencies in G4‐DNA helicases can cause DNA damage, defective cell growth, and various diseases (Ishikawa, 2013; Maizels & Gray, 2013; Mendoza et al., 2016; Varshney et al., 2020). Evidence suggests that helicases that bind to and unwind G4 in vitro may also act directly on G4‐DNA in vivo. For example, the genome‐wide distribution of XPD and PIF1 significantly overlaps with that of G4 motifs (Gray et al., 2014; Paeschke et al., 2011). Green fluorescent protein (GFP)‐tagged PIF1 can form nuclear foci, some of which overlap with the signals of fluorescently‐labeled G4‐binding compounds (Rodriguez et al., 2012).

DHX36 (also known as G4R1 or RHAU) is a member of the DExH/D family of helicases. Helicases are known to bind and rewind both G4‐DNA and G4‐RNA in vitro (Creacy et al., 2008; Vaughn et al., 2005). This protein has much higher binding (Giri et al., 2011) and enzymatic activities (Vaughn et al., 2005) for G4‐DNA compared with the same DNA in an unstructured form. DHX36 participates in various functions including mRNA decay, telomerase regulation, and stress responses, likely by unwinding G4‐RNA (Chalupnikova et al., 2008; Sexton & Collins, 2011; Tran et al., 2004). In addition to its functions via G4‐RNA, DHX36 can also act as a G4‐DNA helicase in vivo (Antcliff et al., 2021; Schult & Paeschke, 2021). Previous reports have suggested that DHX36 targets sequences harboring the G4 motif on gene promoters and regulates their transcriptional activity, possibly by resolving G4‐DNA (Huang et al., 2011; Lai et al., 2012). In vitro DNA replication experiments have shown that DHX36 binds to G4‐DNA and promotes DNA synthesis past G4 (Sato et al., 2021). Extracts from DHX36‐immunodepleted HeLa cells lose more than 50% of their whole‐cell G4‐DNA‐resolving activities (Vaughn et al., 2005). Despite these findings, the in vivo spatial connection between DHX36 and G4‐DNA, and the biological implications of DHX36 as a G4‐DNA helicase, remain unclear. Thus, in the present study, we investigated the in vivo functions of DHX36 and its association with G4‐DNA. We revealed a close connection between DHX36 and G4‐DNA and its relevance to genomic integrity.

## 2 RESULTS

### 2.1 Overlapping DHX36 and G4‐DNA foci in nuclei

**FIGURE 1:**
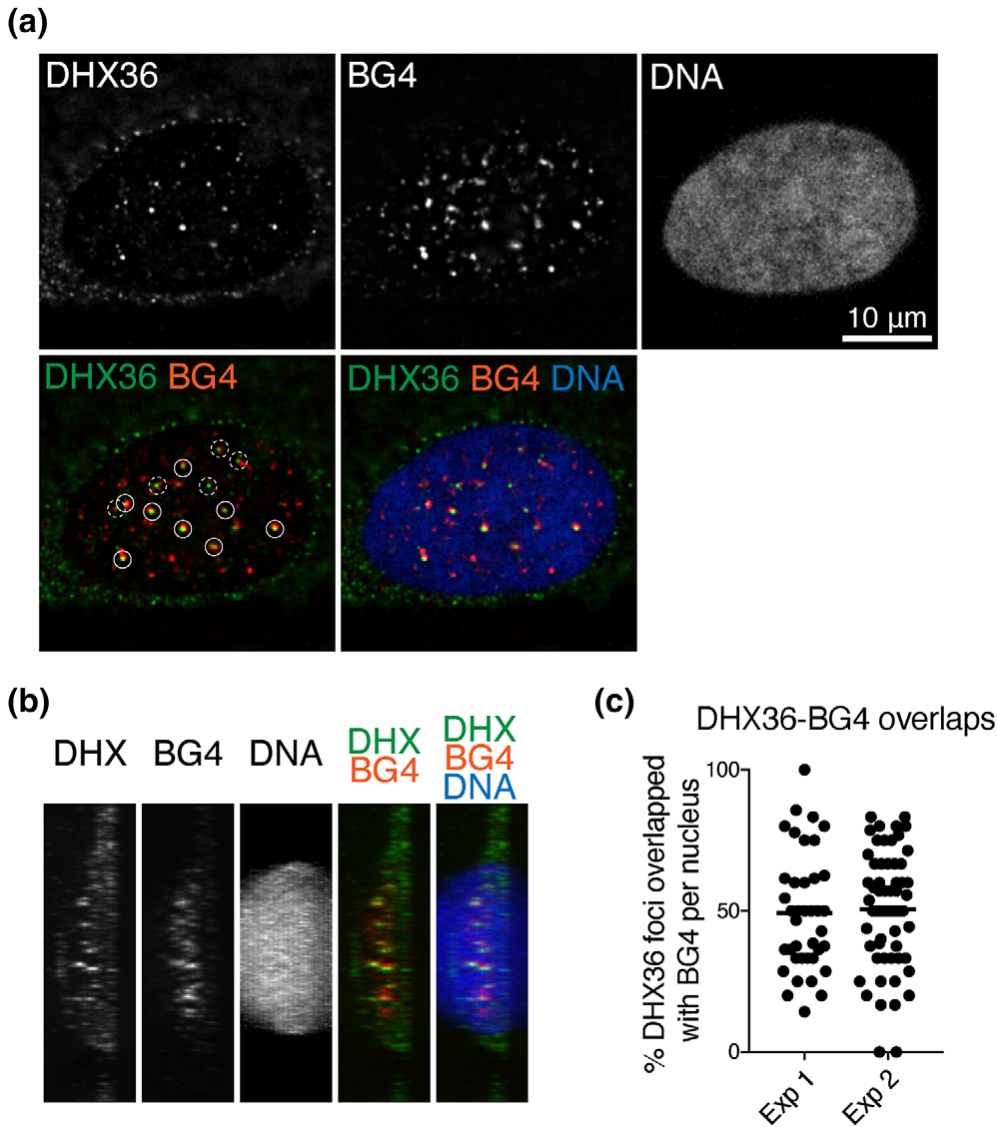
Overlap of DHX36 and BG4 nuclear localization. (a) Deconvoluted images of IMR90 cells immunostained for DHX36 and G4. DNA was counterstained with Hoechst 33342. Among the detected nuclear DHX36 foci, the distinct foci are marked by circles with solid or dotted lines. DHX36 foci overlapping or not overlapping with BG4 foci are marked by circles with solid and dotted lines, respectively. The foci and their overlap were detected using the Image J software. (b) Side view of stacked images of the immunostained cells prepared in (a). (c) 50.0% of nuclear DHX36 foci overlapped with BG4 signals (average from two independent experiments). The number of overlapped DHX36‐BG4 foci over that of distinct nuclear DHX36 foci is represented as percent DHX36–BG4 overlaps.

The subcellular localization of endogenous DHX36 in normal human diploid fibroblasts, IMR90, was analyzed using immunofluorescence staining (IF). DHX36 signals were detected in nuclei and cytoplasm, with some discrete foci visible in the nuclei (Figure 1a). We assessed the specificity of DHX36 staining by comparing the IF signals in cells expressing shRNA targeting DHX36 with those in control cells (Figure S1). The nuclear foci in control cells (Figure S1a, shVector) were largely reduced by DHX36 depletion (Figure S1a, sh*DHX36*), indicating that the signals represent intrinsic DHX36 protein. To test whether nuclear DHX36 associates with G4‐DNA in the cellular context, IF analysis was performed using a BG4 antibody. This single‐chain variable fragment (scFv) antibody has the highest in vitro binding specificity for any form of G4‐DNA and G4‐RNA, but not for non‐G4 nucleotides, including ssDNA, dsDNA, or RNA hairpins (Biffi et al., 2013; Biffi et al., 2014). Notably, nuclear IF signals of BG4 reflect G4‐DNA but not G4‐RNA (Biffi et al., 2013). We observed that BG4 showed discrete nuclear foci, and a significant fraction of these foci overlapped with DHX36 foci (Figure 1a,b). With an average of 7.4 DHX36 foci per nucleus, approximately 50.0% overlapped with BG4 signals (an average of two independent experiments: 49.3%, *n* = 39; 50.6%, *n* = 60; Figure 1c).

**FIGURE 2:**
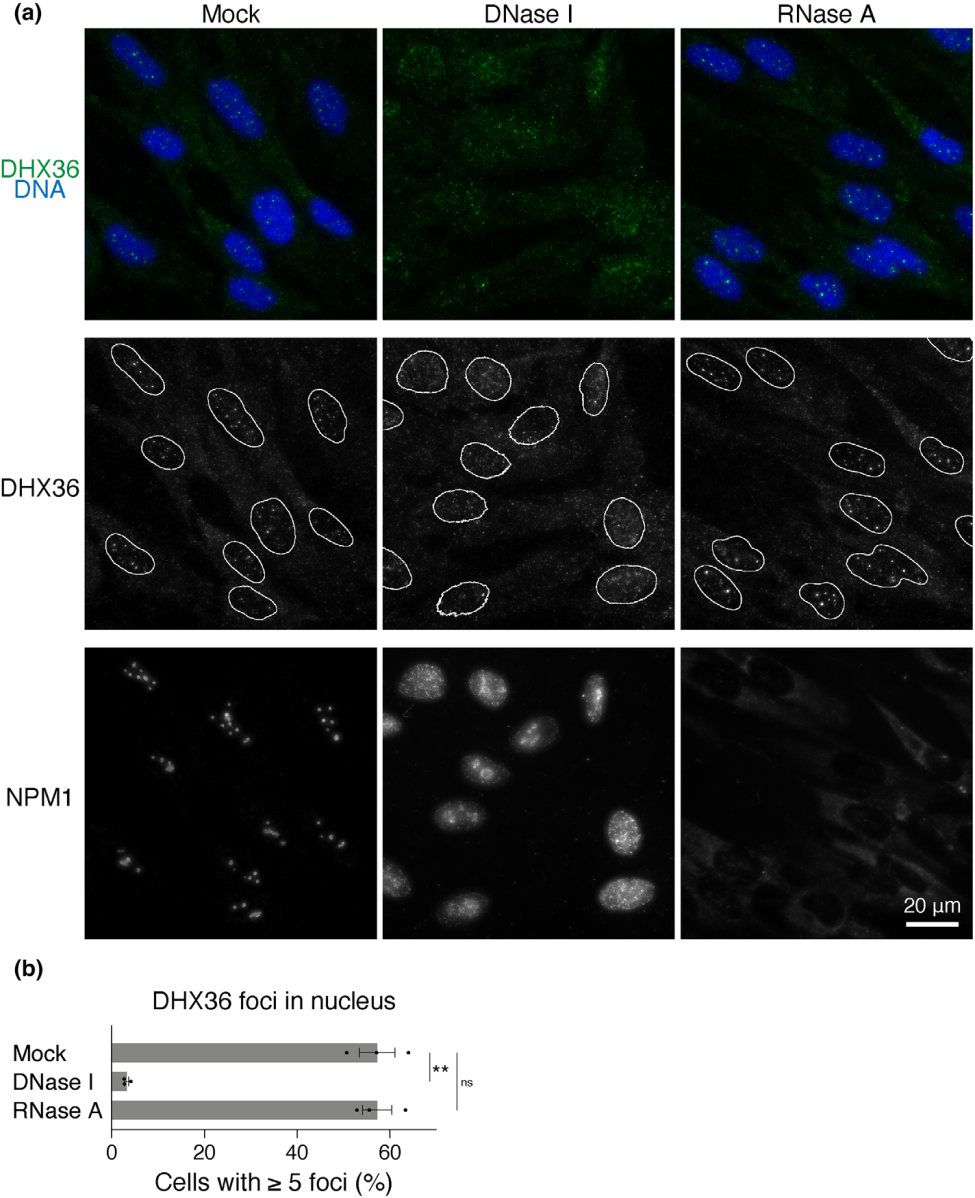
DNA‐dependent formation of DHX36 foci in nuclei. (a) Fixed IMR90 cells were treated with DNase I or RNase A, or were not treated. These cells were immunostained with anti‐DHX36 or anti‐NPM1 antibody. DNA was counterstained with Hoechst 33342. Projection of eight 0.5 μm optical sections through the nucleus and cytoplasm encompassing 3.5 μm is presented. White lines in the middle panels indicate nuclear boundaries. (b) The bar chart describes the percentage of cells with five or more distinct nuclear DHX36 foci among treated or untreated cells as indicated. Values represent mean ± SEM of data from three experiments. Statistical significance of the differences compared to mock‐treated cells was calculated using a paired two‐tailed *t*‐test. **p < .01, ns = not significant.

**FIGURE 3:**
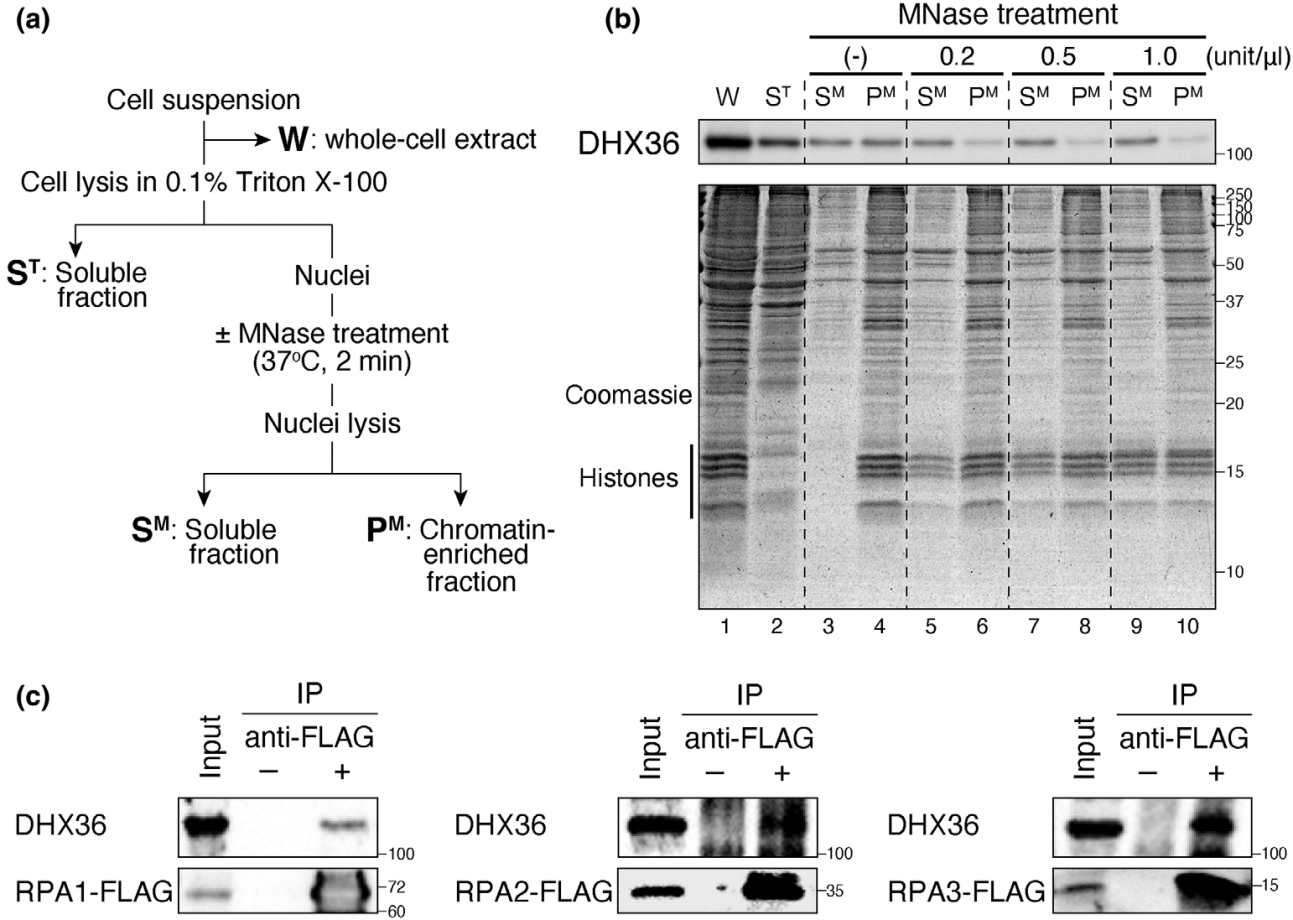
Co‐fractionation of DHX36 and chromatin. (a) Schematic diagram of the subcellular fractionation assay. W, whole‐cell extract; S^T^, soluble fraction after Triton X‐100 treatment; S^M^, soluble fraction after nuclear lysis; P^M^, insoluble pellet after the nuclear lysis. (b) Western blotting of the indicated fractions from IMR90 cells using DHX36 antibody. Isolated nuclear pellets remained untreated or were treated with three different concentrations of MNase. Coomassie staining of the loaded proteins is shown below. (c) DHX36 in the RPA complex. RPA1‐FLAG, RPA2‐FLAG, or RPA3‐FLAG complex from HEK293FT cells was purified using anti‐FLAG antibody‐attached beads, and separated on a gel. The separated proteins were analyzed using western blotting with DHX36 or FLAG antibody.

Consistent with the nuclear BG4 foci representing G4‐DNA that are lost after DNase treatment (Figure S2) (Biffi et al., 2013), DHX36 foci became less distinct after DNase I treatment (Figure 2). In contrast, the distinct nuclear DHX36 foci did not disappear after RNase A treatment, similar to those of BG4 (Figures 2 and S2). More than 50% of the untreated and RNase‐treated cells showed five or more distinct DHX36 foci, whereas DNase I treatment reduced the number of these cells (Figure 2b). DNase and RNase activities were confirmed using IF analysis of nucleophosmin (NPM1), which localizes to the nucleoli and diffuses outside after DNase I or RNase A treatment (Yang et al., 2016). The lack of Hoechst staining confirmed DNase activity. Next, we performed a subcellular fractionation assay to examine whether DHX36 is associated with chromatin (Figure 3a) (Mendez & Stillman, 2000). The cell extract was first separated into soluble (S^T^) and insoluble fractions by Triton X‐100 treatment. The insoluble nuclear pellet was lysed in hypotonic buffer and separated into soluble (S^M^) and insoluble fractions (P^M^), the latter enriched in chromatin and nuclear matrix. Although DHX36 was distributed in all fractions tested (Figure 3b, lanes 2–4), a fraction of DHX36 was distributed in the insoluble fraction (P^M^, lane 4). DHX36 in the P^M^ fraction was released into the soluble S^M^ fraction upon micrococcal nuclease (MNase) treatment of nuclear pellet prior to lysis (lanes 5–10). Because MNase is an endonuclease that digests protein‐free oligonucleotides, these results suggest that DHX36 is associated with chromatin and is not tethered to nuclear skeletal structures.

A comprehensive proteomic study suggests an association between DHX36 and replication protein A (RPA)–ssDNA complex (Maréchal et al., 2014). RPA binds to ssDNA that is formed during DNA replication and transcription (Dueva & Iliakis, 2020), events during which G4‐DNA can be formed. Therefore, we tested whether DHX36 coimmunoprecipitates with RPA. For this, FLAG‐tagged recombinant RPA1, RPA2, or RPA3 was expressed in HEK293FT cells. RPA is an ssDNA‐binding protein complex comprising three tightly associated subunits—RPA1, RPA2, and RPA3 (Dueva & Iliakis, 2020). Cell extracts were divided into two portions: one was mixed with magnetic beads conjugated with Protein A and anti‐FLAG antibody; the other was mixed with magnetic beads conjugated with Protein A but not the antibody. As shown in Figure 3c, DHX36 was enriched in the fraction treated with antibody‐conjugated beads, but not in those without the antibody, in all cases for RPA1, RPA2, and RPA3. The association between DHX36 and RPA1, RPA2, or RPA3 was retained after DNase I treatment (Figure S3), excluding the possibility of RPA–DHX36 aggregation through DNA. Collectively, the results of IF and biochemical analyses imply that DHX36 associates with G4‐DNA in human cells.

### 2.2 DHX36 and G4‐DNA foci overlap with the signals of transcription‐related factors in nuclei

**FIGURE 4:**
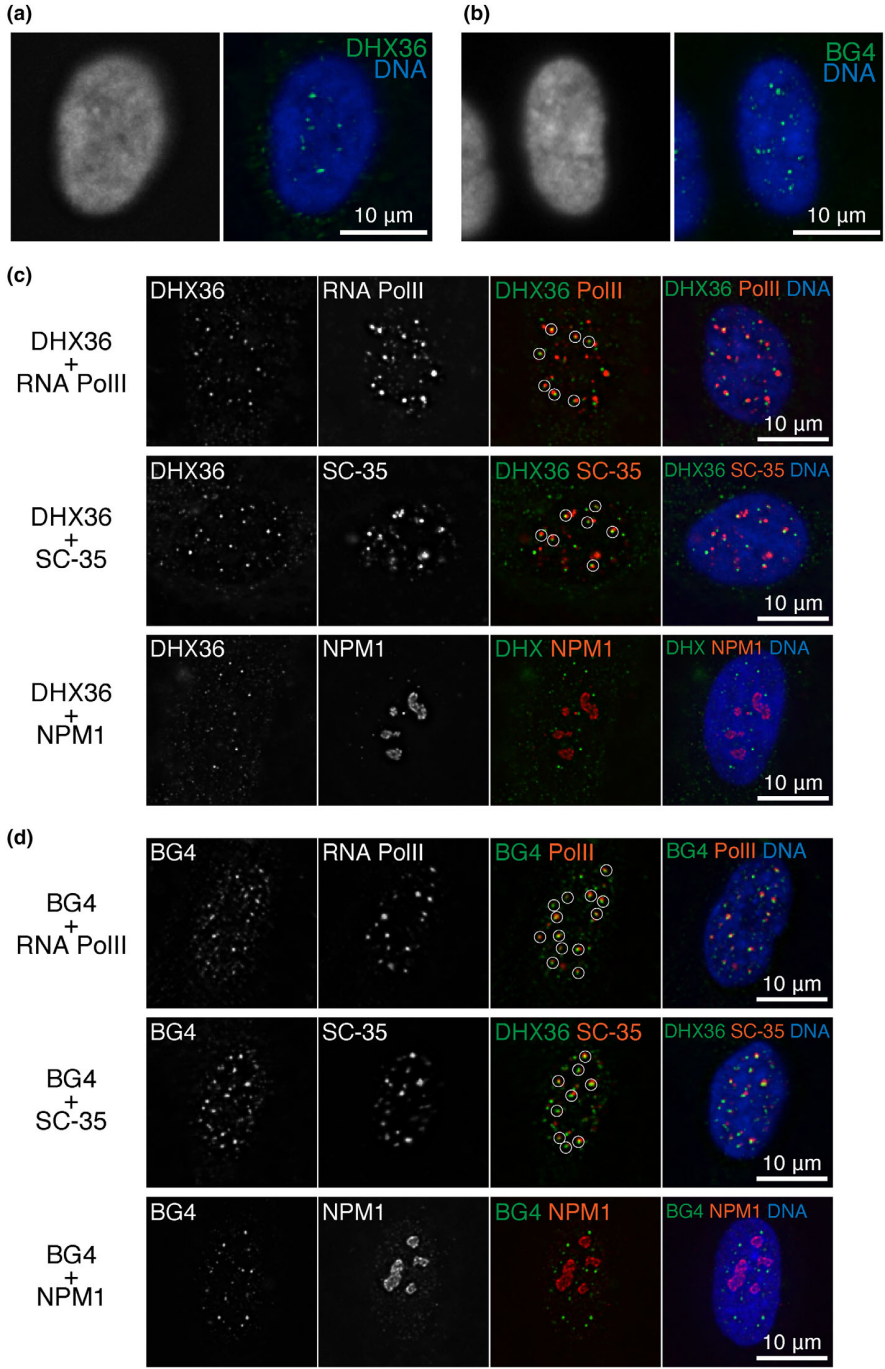
Overlap of DHX36 and BG4 with transcription‐related factors but not with nucleolar proteins in nuclei. (a and b) Nuclear DHX36 (a) and BG4 (b) foci localize to the interspace between compacted chromatin clusters in IMR90 cells. Deconvoluted images from a single optical section of immunofluorescence with the indicated antibodies. (c) Double‐staining of DHX36 and either SC‐35, RNA polymerase II (8WG16), or NPM1. Immunofluorescence and the detection of signal overlaps were performed as described in Figure 1. (d) Immunofluorescence using the indicated antibodies was performed as described in (c). A circle marks overlapping signals.

In IF experiments, we noticed that the nuclear foci of DHX36 and BG4 localized to areas where DNA staining signals were weak (Figure 4a,b). These areas are most likely the interspaces between compacted chromatin clusters in the nucleus, termed the active nuclear compartment (ANC), where transcriptionally active or transcriptionally competent chromatin exists (perichromatin region: PR), or where DNA is absent (interchromatin compartment: IC) (Cremer et al., 2015). ANC is the nuclear subcompartment for several biological activities, including gene transcription and RNA processing. Therefore, we examined the spatial distribution of DHX36 and RNA polymerase II or SC‐35, a splicing component (Figure 4c). Consistent with previous observations showing that the signals of ectopically expressed DHX36 overlap with those of SC‐35 (Iwamoto et al., 2008), the foci of endogenous DHX36 observed in our study also considerably overlapped with those of SC‐35 (Figure 4c, middle). We also observed overlapping signals for DHX36 and RNA polymerase II (Figure 4c, top). Although DNA was generally less stained in the nucleoli, DHX36 signals did not overlap with the nucleoli marked by NPM1 (Figure 4c, bottom). Consistent with our observation that DHX36 and BG4 signals overlapped, the BG4 signals also overlapped with those of RNA polymerase II and SC‐35, but not of NPM1 (Figure 4d), suggesting a role of DHX36 in the clearance of G4, which is related to transcription.

### 2.3 Growth inhibition and elevated DNA damage induction in DHX36‐depleted cells

**FIGURE 5:**
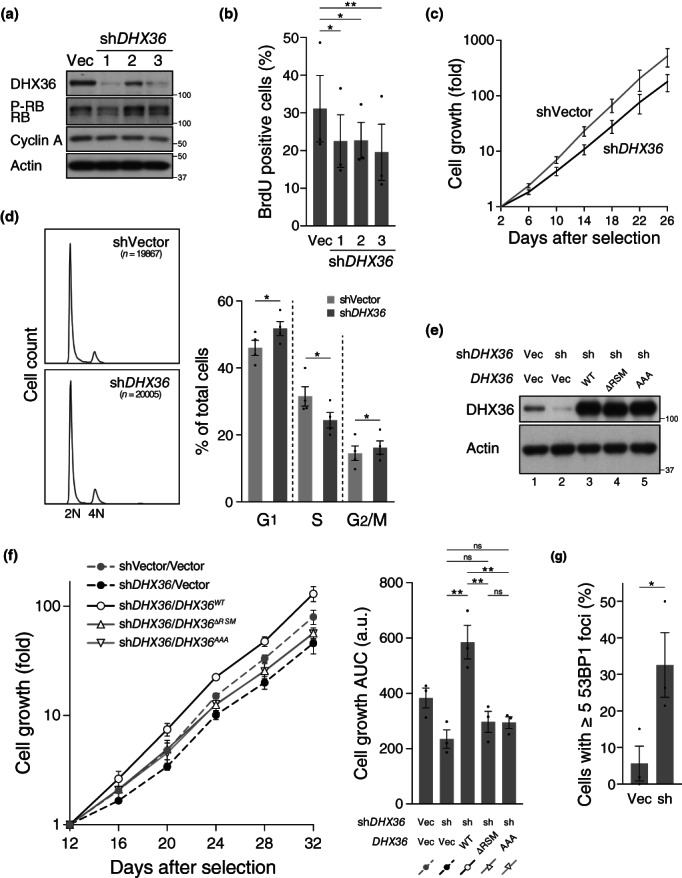
Slower growth and increased DNA damage in DHX36‐depleted cells. (a) Western blot analysis for the indicated antibodies in IMR90 cells expressing three different sh*DHX36*. (b) Reduced BrdU incorporation in sh*DHX36*‐expressing cells. Cells in (a) were assessed for BrdU incorporation. (c) Growth of sh*DHX36*‐expressing cells or control cells was assessed by counting cell numbers once in 4 days after selecting infected cells. (d) Altered distribution of cells in different cell‐cycle stages. Cells stained with propidium iodide (PI) were subjected to flow cytometry analysis, and the relative DNA content was plotted against cell count (left). The distribution of the cells in the indicated stages was analyzed using the Watson model (right). (e and f) The reintroduction of DHX36 rescued the slow‐growth phenotype in the DHX36‐depleted cells. Indicated combinations of vectors were transduced using viral infection. Immunoblot analysis for the indicated proteins in cells ectopically expressing DHX36 variants (e). Cell growth (f) was assessed as in (c). Area under the curve (AUC) was calculated using the cell growth curve shown in the left panel. (g) Increased DNA damage in sh*DHX36*‐expressing cells. Cells were immunostained with an anti‐53BP1 antibody. Cells showing five or more 53BP1 foci were counted as positive. See text for details of DHX36 mutants. Values represent mean ± SEM of data from three or more experiments in all figures. Statistical significance was calculated using a paired two‐tailed *t*‐test (d) or one‐way analysis of variance (ANOVA) with Dunnett's correction (b) or Tukey's correction (f) for multiple comparisons. **p < .01, *p < .05, ns = not significant.

To examine whether DHX36 maintains genomic integrity and cell growth, we generated shRNAs targeting three different sequences on *DHX36* mRNA. The shRNAs exhibited different knockdown efficiencies (Figure 5a). Expression of sh*DHX36‐1* or sh*DHX36‐3* interfered with cell cycle progression, as determined by a reduction in the phosphorylation of RB and 5‐bromo‐2′‐deoxyuridine (BrdU) incorporation (Figure 5a,b), suggesting a role of DHX36 in cell cycle progression. *shDHX36‐2* expression had milder knockdown efficiency but was still sufficient to reduce BrdU incorporation, similar to *shDHX36‐1* or *shDHX36‐3*. The most effective shRNA, sh*DHX36‐1*, was selected for further analyses.

We investigated whether *DHX36* knockdown affected the proliferation of IMR90 cells. To monitor cell proliferation, we counted the number of cells every 4 days for 24 days. Compared with the control cells, cells expressing *DHX36* shRNA grew slowly (Figure 5c). These cells were also analyzed using flow cytometry to determine their cell cycle distribution (Figure 5d, left panels). Using the Watson's model, cells were grouped into three phases, G1, S, and G2/M (Figure 5d, right). Although the difference was moderate, *DHX36* knockdown reduced the number of cells in the S‐phase (those between 2 N and 4 N) and increased the number of cells in the other phases compared with the proportion among control cells. This observation was consistent with reduced RB phosphorylation and BrdU incorporation (Figure 5a,b). To examine whether the growth defects caused by *DHX36* knockdown could be rescued by reintroducing *DHX36*, we transduced *DHX36* expression constructs into DHX36‐depleted cells (Figure 5e,f). All the shRNAs designed in this study target the 3′‐UTR of *DHX36* mRNA so that the expression of the reintroduced *DHX36* should be unaffected by these shRNAs. The cell types exhibited different levels of growth, as represented by the area under the curve (AUC) (Figure 5f, right). For instance, sh*DHX36*‐expressing cells without reintroduced *DHX36* (sh*DHX36*/Vector) exhibited the slowest growth; therefore, their AUC was the smallest among the cell types tested. The slower‐growth phenotype of sh*DHX36*‐expressing cells (shVector/Vector vs. sh*DHX36*/Vector) was alleviated by ectopic expression of DHX36 (sh*DHX36*/Vector vs. sh*DHX36*/*DHX36* ^*WT*^), excluding the possibility of off‐target effects of sh*DHX36* (Figure 5c,f). Notably, the reintroduction of DHX36 expression not only rescued the slow‐growth phenotype by DHX36 depletion, but also promoted cell growth (Figure 5f, shVector/Vector vs. shDHX36/*DHX36* ^*WT*^). This was accompanied by higher protein levels of DHX36 in the “reintroduced” cells compared with the endogenous level in control cells (Figure 5e, lanes 1 and 3). The changes in cell growth rate in DHX36‐depleted and DHX36‐overproducing cells collectively show that DHX36 is involved in cell growth.

In addition to wild‐type DHX36, either of the two mutants, DHX36^ΔRSM^ or DHX36^ΑΑΑ^, was reintroduced into the cells to test if direct binding of DHX36 to G4 may be required for the rescue of cell growth upon DHX36 reintroduction. DHX36^ΔRSM^ lacks a 13‐amino acid stretch (54–66 aa), termed the RHAU‐specific motif (RSM) (Chalupnikova et al., 2008), which is essential for G4 binding and is, therefore, required for G4 unwinding activity in vitro (Lattmann et al., 2010). A recent structural study of the DHX36–G4 complex revealed that residues G59, I62, G63, and A67 in and around the RSM are responsible for the direct recognition of the four guanine bases in the terminal G‐quartet layer, and K58, R60, and K69 interact with the G4 phosphate backbone (Heddi et al., 2015). DHX36^ΑΑΑ^ includes G59A/I62A/G63A replacements in the RSM. The rescue of cell growth by reintroduction of either *DHX36* ^*ΔRSM*^ or *DHX36* ^*ΑΑΑ*^ was marginal compared with that achieved with *DHX36* ^*WT*^ (Figure 5f, sh*DHX36*/*DHX36* ^*WT*^ vs. sh*DHX36*/*DHX36* ^*ΔRSM*^ or sh*DHX36*/*DHX36* ^*ΑΑΑ*^), albeit with comparable protein levels among the DHX36 proteins (Figure 5e, lanes 3–5), indicating that the rescue of slower cell growth by reintroduction of DHX36 requires the DHX36–G4 interaction.

Next, we examined whether DHX36 suppresses the induction of DNA damage. For this, we counted the number of nuclear signals of 53BP1, which is recruited to DNA double‐strand break (DSB) sites and is, therefore, a widely used DSB marker (Panier & Boulton, 2014), to assess the incidence of DNA damage after *DHX36* knockdown. In cells expressing sh*DHX36*, the percentage of cells with five or more 53BP1 foci was approximately 30%, whereas that in control cells was approximately 5% (Figure 5g), indicating an elevated induction of DNA damage in DHX36‐depleted cells.

### 2.4 DHX36 depletion sensitizes cells to G4‐stabilizing compounds

**FIGURE 6:**
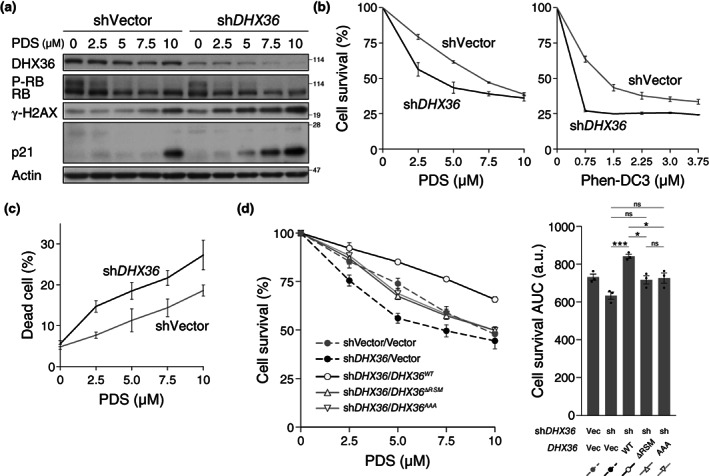
*DHX36* knockdown sensitizes cells to G‐quadruplex‐stabilizing compounds. IMR90 cells expressing sh*DHX36* or control cells harboring shVector were treated with multiple concentrations of PDS or Phen‐DC3 for 2 days. (a) Western blotting with the indicated antibodies for whole‐cell extracts. (b) Cell viability was determined by measuring the dehydrogenase activity in living cells after PDS (left) or Phen‐DC3 (right) treatment. (c) Dead cells were stained with trypan blue after PDS treatment. (d) The reintroduction of DHX36 rescued the elevated sensitivity against PDS in DHX36‐depleted cells. The same assay as in (b) was performed after PDS treatment. Indicated sh‐insensitive *DHX36* variants were expressed under viral promoters using a pMXs vector. AUC was calculated using the cell survival curve shown in the left panel. In all figures, values represent mean ± SEM of data from three experiments. Statistical significance was calculated using one‐way ANOVA with Tukey's correction for multiple comparisons. ***p < .001, *p < .05, ns = not significant.

The G4 structure is dynamic and can be formed when the G4‐forming DNA sequences become single‐stranded during replication or transcription (Maizels, 2015). G4, in turn, interrupts the progression of the replication and transcription machinery, which can result in a DNA gap or break. Stabilization of G4 by treatment of cells with the G4‐stabilizing chemical compound, pyridostatin (PDS) (Rodriguez et al., 2012), leads to an increase in G4‐DNA in nuclei (Biffi et al., 2013), the emergence of DNA damage in a DNA replication‐ and transcription‐dependent manner, and cell cycle arrest (Rodriguez et al., 2012). As we observed that *DHX36* knockdown induced a DNA damage response and inhibited cell growth, we hypothesized that DHX36 depletion could increase the chance of G4 trapping by G4‐stabilizing compounds. Therefore, we investigated whether the inhibitory effect of the G4‐stabilizing compound on cell growth or genome maintenance was mediated by a reduction in cellular DHX36 levels. As reported previously (Rodriguez et al., 2012), higher doses of PDS induced DNA damage and cell cycle arrest, represented by increased γ‐H2AX and p21 levels, respectively (Figure 6a, shVector, 10 μM). These changes were induced at lower concentrations in DHX36‐depleted cells (Figure 6a, sh*DHX36*, 5–10 μM). The survival rate of DHX36‐depleted cells, monitored by determining the respiratory activity of living cells, declined at lower doses of PDS than in control cells (Figure 6b, left). To confirm that G4 stabilization caused cell growth, we tested the effect of another G4 stabilizer, Phen‐DC3 (De Cian et al., 2007), on cell survival. Growth inhibition by the alternative G4 stabilizer was enhanced by DHX36 knockdown (Figure 6b, right). Consistently, the percentage of dead cells was elevated at lower doses of PDS in DHX36‐depleted cells compared with that in control cells (Figures 6c and S4). Collectively, these results indicate that the reduction of DHX36 sensitizes cells to G4‐stabilizing compounds.

We further explored the possibility that altered G4 levels enhance PDS sensitivity in DHX36‐depleted cells. To this end, we transduced expression constructs for *DHX36* or its mutants into DHX36‐depleted cells and checked whether the reintroduction of *DHX36* rescued the elevated PDS sensitivity caused by DHX36 reduction. The enhanced PDS sensitivity in sh*DHX36*‐expressing cells was alleviated by the ectopic expression of DHX36 (sh*DHX36*/Vector vs. sh*DHX36*/*DHX36* ^*WT*^), again excluding the possibility of an off‐target effect of sh*DHX36* (Figure 6d). In contrast, the reintroduction of either *DHX36* ^*ΔRSM*^ or *DHX36* ^*ΑΑΑ*^ failed to reduce the PDS hypersensitivity at the level of *DHX36* ^*WT*^ reintroduction (Figure 6d, sh*DHX36*/*DHX36* ^*WT*^ vs. sh*DHX36*/*DHX36* ^*ΔRSM*^ or sh*DHX36*/*DHX36* ^*ΑΑΑ*^), showing that the rescue of cell survival during PDS treatment by the reintroduction of DHX36 requires the interaction of DHX36 with G4.

Similar to the effect on cell growth in the absence of the compounds (Figure 5f), reintroduction of DHX36 resulted in stronger resistance against PDS than in control (Figure 6d, sh*DHX36*/*DHX36* ^*WT*^ vs. shVector/Vector). Again, this correlated with higher protein levels of DHX36 in the “reintroduced” cells (Figure 5e, lanes 1 and 3). Although expression of DHX36^ΔRSM^ or DHX36^ΑΑΑ^ failed to alleviate PDS sensitivity to the level achieved by its wild‐type counterpart, it conferred PDS resistance to some extent (Figure 6d, sh*DHX36*/Vector vs. sh*DHX36*/*DHX36* ^*ΔRSM*^ or sh*DHX36*/*DHX36* ^*ΑΑΑ*^). This result suggests that these mutants retain G4 unwinding activity despite their reduced direct G4‐DNA binding. We confirmed the enhanced PDS resistance conferred by the overexpression of DHX36 in IMR90 cells without sh*DHX36* (Figure S5).

## 3 DISCUSSION

DHX36 binds to and unwinds G4‐DNA and G4‐RNA in vitro (Creacy et al., 2008; Vaughn et al., 2005). Although based on previous reports, it was surmised that DHX36 targets G4‐DNA and G4‐RNA in cultured cells (Mendoza et al., 2016), its role in processing G4‐DNA is poorly demonstrated, in contrast to its function as a G4‐RNA helicase. Here, we demonstrate that downregulation of DHX36 helicase leads to accumulation of DNA damage, causes slower cell growth, and significantly sensitizes cells to treatment with G4‐stabilizing compounds, which is accompanied by a DNA damage response. DHX36 colocalizes with G4‐DNA in specific regions of the nucleus and is associated with chromatin. Thus, based on our results, together with previous reports showing the G4‐DNA helicase activity of DHX36, we propose that clearance of G4 by DHX36 prevents DNA damage, ensuring genomic integrity.

DHX36 and G4‐DNA colocalize in particular regions of the nucleus where transcription or splicing can occur (Figures 1 and 4). Similar to the nuclear IF signals visualized using BG4 antibody, which represent G4‐DNA (Biffi et al., 2013), nuclear DHX36 foci were less distinct after DNase treatment but not after RNase treatment (Figure 2). The subcellular fractionation assay showed association of chromatin with DHX36 (Figure 3a,b). In in vitro assays, a direct binding of DHX36 to G4‐DNA was observed (Creacy et al., 2008; Giri et al., 2011; Vaughn et al., 2005). Moreover, structural studies indicated that RSM and the oligonucleotide/oligosaccharide‐binding (OB) fold of DHX36 can associate directly with the top of DNA G‐quartet stacks and the surrounding single‐stranded segment (Chen, Tippana, et al., 2018; Heddi et al., 2015). Finally, comprehensive G4 mapping in cells showed that the G4‐binding domain of DHX36 is enriched at G4 (Zheng et al., 2020). Thus, our data, together with the previous findings, imply an association between DHX36 and G4‐DNA in human cells. Although the precise mechanism by which DHX36 is recruited to the G4‐forming region in vivo remains unknown, our observation of the association between DHX36 and the RPA complex (Figure 3c) suggests that the unwinding of dsDNA and the resulting ssDNA formation may promote DHX36 recruitment to chromatin. This possibility is supported by a previous comprehensive proteomic study that showed an association between DHX36 and the RPA−ssDNA complex (Maréchal et al., 2014). These observations and the potential of direct binding between DHX36 and ssDNA (Chen, Tippana, et al., 2018; Giri et al., 2011) suggest that DHX36 can be recruited to G4‐DNA through ssDNA formed in proximity to G4 or on its complementary strand.

Although the complete picture of G4 dynamics in vivo remains elusive, it has been proposed that the chance of G4 formation increases when ssDNA is created. These situations include transcription, DNA replication, and repair processes. Recent studies have revealed the prevalence of G4‐DNA in the regulatory regions of actively transcribed genes and nucleosome‐free sites (Hänsel‐Hertsch et al., 2016; Spiegel et al., 2021; Zheng et al., 2020). Furthermore, G4‐DNA signals were observed by increase in IF in cells in the S phase compared with those in quiescence or at the G1/S border (Biffi et al., 2013). Thus, G4 formation may be dynamically regulated, although stable G4 should also be present throughout the chromosomes (Henderson et al., 2013). Our IF results suggest that DHX36 is involved in regulating the formation of G4‐DNA during transcription. Nuclear DHX36 and BG4 signals overlapped with the RNA polymerase II and SC‐35 signals, which are markers of transcription and splicing speckles, respectively (Figure 4c,d). Splicing occurs co‐transcriptionally (Herzel et al., 2017) and speckles are believed to be free from DNA, providing components involved in splicing at sites where transcription and splicing occur (Cremer & Cremer, 2001; Lamond & Spector, 2003; Misteli et al., 1997). Therefore, DHX36 is presumably not a component of the speckles but rather localizes in proximity to them and is associated with transcription. Highly transcribed chromosomal domains are located close to the speckles (Chen, Zhang, et al., 2018; Ishov et al., 2020). Additionally, there is a special connection between active transcription and G4‐DNA formation (Varshney et al., 2020). These reports support our observation of an overlap between G4 and RNA polymerase II or SC‐35 in nuclei (Figure 4d). A recent report suggested the supportive function of DHX36 in DNA replication (Sato et al., 2021). Our observation of increasing nuclear DHX36 signals in the S‐phase (Figures S6 and S7), along with similar results for BG4 (Biffi et al., 2013), also suggests that DHX36 plays a crucial role in DNA replication. Despite this, we propose that DHX36 might contribute not only to replication but also to other functions, such as transcription, as its depletion induces a reduction, but not an increase, in the proportion of S‐phase cells (Figure 5d). Several studies have demonstrated transcriptional regulation by DHX36 (Huang et al., 2011; Lai et al., 2012). Moreover, the RPA complex, which can bind to DHX36 (Figure 3c), localizes to a region where transcription occurs, possibly through its association with ssDNA displaced in R‐loops (Dueva & Iliakis, 2020; Zhang et al., 2017). Precise mapping of DHX36 on chromatin will help reveal its target site and predict its biological functions.

Treatment with G4‐binding compounds induces accumulation of DNA damage and causes cell cycle arrest (Rodriguez et al., 2012). Because the induction of DNA damage is replication‐ and transcription‐dependent, the collision between stabilized G4‐DNA and the replication or transcription machinery appears to produce DNA breaks. Our observation that DHX36 depletion enhanced the sensitivity of cells to PDS or Phen‐DC3 and increased the incidence of DNA damage suggested that G4‐binding compounds may trap the remaining G4 accumulated in DHX36‐depleted cells. Binding modes of DHX36 and PDS against G4 are reportedly similar (Balasubramanian et al., 2011; Chen et al., 2015; Heddi et al., 2015; Rodriguez et al., 2012) and DHX36 shows a remarkably higher affinity for G4 (Creacy et al., 2008), raising the possibility that competitive and stable binding of DHX36 may interrupt PDS binding to G4. Moreover, this scenario can explain our observation that the overexpression of DHX36 confers resistance to PDS treatment (Figure 6d). However, this is unlikely because the highly expressed DHX36 does not inhibit but promotes cell growth (Figure 5f), indicating that DHX36 associates with G4‐DNA transiently and precludes G4 trapping by PDS by resolving the structure. Although we cannot exclude the possibility that DHX36 engages in the maintenance of genomic integrity indirectly by targeting G4‐RNA, our data imply that DHX36 suppresses the induction of DNA damage by targeting and resolving G4‐DNA. Deficiency of other helicases with G4‐binding and unwinding activities, such as BLM, RTEL1, WRN, PIF1, and DNA2, causes DNA damage at putative G4 sites (Crabbe et al., 2004; Lin et al., 2013; Paeschke et al., 2011; Sfeir et al., 2009; Vannier et al., 2012). Although the enzymatic activity of some helicases is unlikely to be specific to G4 and, therefore, it may be difficult to distinguish between their activity against G4 and other substrates, it is crucial to address how the target G4‐DNA is separated or shared among G4 helicases. Some studies have shown different substrate preferences (G4 topologies) of G4 helicases (Mendoza et al., 2016). Nevertheless, when or where these helicases differentially work on chromosomes remains an open question.

Our data show that the upregulation of DHX36 protein levels promotes cell proliferation and confers resistance to treatment with G4‐stabilizing compounds, whereas its depletion induces the opposite effects (Figures 5 and 6). DHX36 binds to and unwinds the G4‐RNA on telomerase RNA and maintains telomere length, possibly by regulating its catalytic activity (Booy et al., 2012; Sexton & Collins, 2011). As telomere maintenance by telomerase is required for continuous cell proliferation, DHX36 may promote cell proliferation through telomere maintenance. However, we propose that DHX36 regulates cell proliferation through mechanisms other than telomerase regulation because the cells used in this study are normal fibroblasts that do not exhibit telomerase activity (Kim et al., 1994). It is still debated, but increasing evidence has shown that G4 directly regulates gene transcription either positively or negatively. Previous reports have shown that G4 clearance from the promoters of cell growth‐related genes by helicases, including DHX36, is required for the transcription of these genes (Mendoza et al., 2016). Therefore, DHX36 may affect cell growth by modulating promoter G4 structure and its downstream gene transcription. However, we propose a different perspective. Our data showed that DHX36 depletion enhanced DNA damage induction, growth inhibition, and cell death caused by treatment with G4 stabilizers (Figures 5 and 6). Because these detrimental effects are induced in a transcription‐ and replication‐dependent manner (Rodriguez et al., 2012), we propose that DHX36 aids cell growth by maintaining genomic integrity by scavenging G4‐DNA during these events.

## 4 EXPERIMENTAL PROCEDURES

### 4.1 Cell culture and gene transfer

IMR90 human lung fibroblasts were used, unless otherwise stated. HEK293FT cells were used for immunoprecipitation analysis. Cells were cultured in Dulbecco's modified Eagle medium supplemented with 10% fetal bovine serum (FBS). Retroviral gene transfer was performed as described (Narita et al., 2003) except that Plat‐A cells were used for viral packaging (Morita et al., 2000) and FuGENE HD (Promega, E2311) or PEI MAX (Polysciences, 24765‐1) was used for transfection. Quiescence (G0 phase) was induced by incubating cells in DMEM with 0.1% FBS for 2–3 days.

### 4.2 Plasmids

The following retroviral plasmids were used: pMXs‐*DHX36* (puro), pMXs‐*DHX36* ^*ΔRSM*^ (puro), and pMXs‐*DHX36* ^*AAA*^ (puro). *DHX36* cDNA (NCBI: CCDS3171) was cloned using the WI38/*hTERT* mRNA. Deletion of RHAU‐specific motif (RSM: 54–66 aa), a conserved motif among DHX36 orthologs, and introduction of AAA mutations (G59A/I62A/G63A) were conducted using PCR (Imai et al., 1991) with following primers: DHX36‐RSM_Rv (5′‐ATGCCGGCCCCTGCCGCCTCGA‐3′) and DHX36‐delRSM_Fw (5′‐GCGAAAAAACAGGGGCAGAAGAAC‐3′) for deletion, DHX36‐RSM_Rv and DHX36‐G59A/I62A/G63A_Fw (5′‐CCCGGGCACCTGAAAGCCCGCGAAGCCGCCATGTGGTACGCGAAAAAACAG‐3′) for AAA mutation. miR30‐based short hairpin RNA was expressed from pMSCV (puro) or pMSCV (hygro) (Silva et al., 2005). The following sequences were designed for shRNA targeting against DHX36: 5′‐GCCATTCTTCATCATTGTT‐3′ (#1), 5′‐CCAAACCCTGGGACATGAA‐3′ (#2), and 5′‐GACTTAATGTGCATGACTT‐3′ (#3). The expression vector for BG4 (pSANG10‐3F‐BG4) was gifted by Shankar Balasubramanian (Addgene Plasmid #55756) (Biffi et al., 2013). hRPA1_1Flag_pCMV6AC, hRPA2_1Flag_pCMV6AC, and hRPA3_1Flag_pCMV6AC were used for immunoprecipitation.

### 4.3 Antibodies

The following antibodies were used: DHX36 (ab70269, Abcam; RN113PW, MBL), RB (554136, Becton Dickinson), γ‐H2AX (05‐636, EMD Millipore), p21 (sc‐817, Santa Cruz Biotechnology), Actin (MAB1501R, EMD Millipore), 53BP1 (NB100‐304, Novus Biologicals), Cyclin A (sc‐751, Santa Cruz Biotechnology), Cyclin B1 (sc‐245, Santa Cruz Biotechnology), PCNA (sc‐56, Santa Cruz Biotechnology), FLAG (F3165 or F7425, Sigma‐Aldrich; MBL, M185‐3L), RNA polymerase II (920101, BioLegend), SC‐35 (ab11826, Abcam), and NPM1 (ab86712, Abcam). Dynabeads Protein G (Invitrogen, 10003D) was used for immunoprecipitation. For BG4 preparation (Figure S8), *E. coli* BL21 (DE3) codon plus (230240, Agilent Technologies) was transformed with pSANG10‐3F‐BG4. BG4 expression was induced by adding 1 mM IPTG to the culture of the transformants, and the cells were lysed with an extraction buffer (50 mM sodium phosphate, pH 7.0, 300 mM NaCl, 0.5% NP‐40, and 1 mM PMSF). After sonication to solubilize the proteins, TALON metal affinity resin (635502, Becton Dickinson) was added to collect the 6× His‐tagged BG4. The resin was washed with wash buffer (50 mM sodium phosphate, pH 7.0, 300 mM NaCl, and 5 mM imidazole) in a Mini‐Column M (Muromachi Chemicals), and bound BG4 was eluted and collected with elution buffer (50 mM sodium phosphate, pH 7.0, 300 mM NaCl, and 150 mM imidazole). Purified BG4 was dialyzed against PBS to replace the buffer.

### 4.4 Cell growth and viability assays

The cells were treated with PDS (SML0678, Sigma‐Aldrich) or Phen‐DC3 (26000, Polysciences) for 2 days. Cell survival was measured using the Cell counting kit‐8 (CK04, Dojindo), and dead cells were stained with trypan blue. For the cell growth assay, cells were counted using a Coulter counter (Beckman Coulter), and 1 × 10^6^ cells were plated on 10 cm plates every 4 days.

### 4.5 Immunofluorescence, microscopic, and quantitative analyses

Immunofluorescence analysis was performed as described (Narita et al., 2003). Cells grown on coverslips were fixed in methanol or methanol/acetic acid (3:1) for 10 min. DNA was counterstained with Hoechst 33342. For G4 staining, fixed cells were incubated at room temperature for 45 min with the following antibodies: primary antibodies (150 ng/mL purified BG4 for mouse and 37 ng/mL for rabbit anti‐FLAG antibodies), secondary antibodies (20 μg/mL for mouse and 8 μg/mL for rabbit anti‐FLAG antibodies), and fluorescence‐conjugated tertiary antibodies. Images were acquired using a DeltaVision Elite imaging system (GE Healthcare) equipped with a wide‐field fluorescence microscope (IX71, Olympus) and a CCD camera (CoolSNAP HQ^2^, Photometrics), and processed using the SoftWoRx software. The number of foci signals per nucleus or the frequency of signal overlap was counted after the IF images were processed using the ImageJ software (https://imagej.nih.gov/ij/) with or without the colocalization plugin. To perform cell cycle analysis, images of cells stained with anti‐PCNA antibodies and Hoechst 33342 were captured using a fluorescence microscope BZ‐X710 (Keyence). The signal intensity of PCNA or Hoechst staining per nucleus was measured using the Hybrid Cell Count application (Keyence). For enzyme treatments, cells were incubated with 0.12 U/μL Turbo DNase (AM2238, Ambion) or 50 μg/μL RNase A (Nippon gene, 312‐01931) at 37°C for 1 h after fixation. Phase‐contrast images of the cells in culture dishes were acquired using an IMT2 microscope (Olympus) equipped with a Pro 600ES camera (Pixera).

### 4.6 Subcellular fractionation assay

Subcellular fractionation assay was performed as described, with some modifications (Mendez & Stillman, 2000). Cells (5 × 10^6^) were washed once with PBS and then resuspended in 260 μL Buffer A (10 mM HEPES‐KOH (pH 7.9), 10 mM KCl, 1.5 mM MgCl_2_, 0.34 M sucrose, 10% glycerol, 1 mM DTT, 1 mM PMSF, protease inhibitor cocktail Complete EDTA‐free (05056489001, Roche), phosphatase inhibitor cocktail PhosSTOP (4906837001, Roche); 60 μL suspension was removed as whole‐cell extract (W). Triton X‐100 (0.1%) was added to lyse cells on ice for 5 min. After centrifugation (3500 rpm, 5 min, 4°C), the supernatant was kept as a soluble fraction (S^T^), and the nuclear pellet was washed once with 200 μL Buffer A. The nuclei were resuspended in 200 μL Buffer A, and CaCl_2_ was added (1 mM). The nuclei suspension was divided into four 50 μL aliquots, and a micrococcal nuclease (MNase, 2910A, Takara) was added (0, 0.2, 0.5, and 1 U/μL). After 2 min of incubation at 37°C, EGTA (1 mM) was added to terminate the MNase reaction. Nuclei were collected by centrifugation (3500 rpm, 5 min, 4°C) and were lysed in 50 μL Buffer B (3 mM EDTA [pH 8.0], 0.2 mM EGTA [pH 8.0], 1 mM DTT, 1 mM PMSF, protease inhibitor cocktail, phosphatase inhibitor cocktail) on ice for 30 min. After centrifugation (3500 rpm, 5 min, 4°C), the supernatant was removed as a soluble fraction (S^M^). The chromatin pellet (P^M^) was washed once with Buffer B and lysed in the SDS‐PAGE sample buffer.

### 4.7 Immunoprecipitation assay

Immunoprecipitation assay was carried out as described (Miyoshi et al., 2019) except that HEK293FT cells, PEI MAX for transfection, and Lysis 150 buffer (20 mM Tris–HCl (pH 8), 2.5 mM MgCl_2_, 150 mM KCl, 0.5% NP‐40, 1 mM DTT, 1× cOmplete EDTA‐free protease inhibitor cocktail (Roche, 11873580001), 0.2 mM PMSF) were used. For immunoprecipitation of RPA1 or RPA3, cells grown on two 10 cm dishes were used for preparing cell extracts, and 30 μL of eluent was obtained. For RPA2, cells were grown on four 10 cm dishes. Immunoprecipitation was performed with a two‐times scale compared with the case for RPA1 and RPA3, and 30 μL of eluent was obtained. For DNase treatment, the precipitated complex was incubated with DNase buffer (20 mM Tris–HCl [pH 8], 5 mM MgCl_2_, 50 mM NaCl, 1 mM DTT) containing 250 U/μL DNase I (2270A, Takara) at 37°C for 30 min.

### 4.8 Flow cytometry analysis

Cells (2 × 10^6^) were plated onto a 10‐cm dish the day before sample preparation. Cells were trypsinized, collected, and washed with FACS buffer (PBS containing 0.5% FBS and 0.1% sodium azide). The cells were then fixed with 70% ethanol (ethanol diluted with FACS buffer) and treated with RNase solution (FACS buffer with 5 μg/mL RNase (Nippon Gene, 313–01461)) for 30 min at 37°C. The cells were filtered through a nylon mesh (N‐No. 150 T), transferred to a round‐bottom tube (Falcon, 352,008), treated with 50 μg/mL propidium iodide (PI), and analyzed with FACSCalibur (BD Biosciences) equipped with FlowJo (BD Biosciences). Cells (~2 × 10^4^) were analyzed to obtain the cell cycle distribution pattern.

### 4.9 Statistical analysis

Statistical analyses were conducted using Prism 7 (GraphPad). Differences with ***p < .001, **p < .01, and *p < .05 were considered significant.

## AUTHOR CONTRIBUTIONS

Ayaka Mizumoto, Fuyuki Ishikawa, and Mahito Sadaie conceived the study. Ayaka Mizumoto, Yuta Yokoyama, Tomoichiro Miyoshi, Masahiro Takikawa, and Mahito Sadaie designed and performed the experiments. Ayaka Mizumoto, Yuta Yokoyama, and Mahito Sadaie wrote the original manuscript. Tomoichiro Miyoshi, Masahiro Takikawa, Fuyuki Ishikawa, and Mahito Sadaie reviewed the manuscript.

## CONFLICT OF INTEREST STATEMENT

The authors declare that they have no conflicts of interest regarding the contents of this article.

## Supporting information


**Figure S1.** Validation of DHX36 antibodies. (a) Immunofluorescence staining of IMR90 cells transduced with sh*DHX36* expression vector or control vector using anti‐DHX36 antibodies. Projection of 13 0.75 μm optical sections through the nucleus and cytoplasm encompassing 9 μm. (b) Western blotting analysis using indicated antibodies for cells used in (a).
**Figure S2.** Loss of nuclear BG4 foci after DNase treatment. Fixed IMR90 cells were treated with DNase I or RNase A, or were left untreated. These cells were immunostained with BG4 antibodies.
**Figure S3.** DHX36–RPA complex is retained after DNase I treatment. RPA1‐FLAG, RPA2‐FLAG, or RPA3‐FLAG complex from HEK293FT cells was purified using anti‐FLAG antibody‐attached beads. After subsequent treatment of the beads‐attached complex with DNase I (250 U/μL), proteins were separated on a gel and analyzed using western blotting with DHX36 or FLAG antibody.
**Figure S4.** Reduced survival under PDS treatment in DHX36‐depleted cells. IMR90 cells expressing sh*DHX36* or control cells harboring shVector were treated with multiple concentrations of PDS for 2 days. Representative images of the cells in the experiment in Figure 6a–c are shown.
**Figure S5.** DHX36 overproduction desensitizes cells to G4 stabilizers. IMR90 cells ectopically expressing DHX36 from viral promoters using a pMXs vector were treated with multiple concentrations of PDS for 2 days. (a) Cell viability was determined by measuring the dehydrogenase activity of living cells after PDS treatment. Values represent mean ± SEM of data from three experiments. (b) Western blotting with the indicated antibodies for whole‐cell extracts.
**Figure S6.** Cells in S‐phase are represented by their higher PCNA signal intensity in the nuclei. (a) Images of IMR90 cells stained with PCNA antibodies and Hoechst 33342 (DNA). Arrowheads indicate nuclei with higher PCNA signal intensity. (b) The signal intensity of PCNA or Hoechst staining per nucleus was calculated using the images in a. Nuclei were plotted according to PCNA and Hoechst signal intensities (*n* = 3171). (c) Cell cycle profiles of cells in b. Populations with DNA content over 4n are likely to be clumping cells on a coverslip (b and c).
**Figure S7.** Nuclear DHX36 and BG4 signals increase in the S‐phase. (a) Representative images of nuclei stained with DHX36 and PCNA antibodies. S‐phase nuclei were identified by their higher PCNA intensities. (b) Average of nuclear foci of G4 or DHX36 in the S‐phase or non‐S‐phase. At least 30 nuclei were counted for each stage. (c) DHX36 protein levels remained unchanged throughout the cell cycle, as seen using western blotting with the indicated antibodies for whole‐cell extracts. Cells were synchronized in the G0 phase by incubating with low‐serum media for 2–3 days and released into the cell cycle for the period shown above. Values are represented as mean ± SEM of data from three experiments. Statistical significance was calculated using two‐tailed paired t‐tests. **p < .01, * *p* < .05. Asy, asynchronous cells.
**Figure S8.** Expression and purification of BG4 scFv. (a) Whole‐cell extracts of Escherichia coli cells harboring pSANG10‐3F‐BG4 with or without IPTG‐induced expression of 6 × His‐tagged BG4 single‐chain variable fragment (scFv). (b) His‐tagged BG4 scFv was collected using a metal affinity resin and eluted in a buffer containing imidazole. (c) Eluate fractions No. 5 and 6 from b were combined and dialyzed against PBS buffer. This purified BG4 scFv was used for immunofluorescence. All the gels were stained with Coomassie Brilliant Blue.
