# Supplementary material for: DHX36 maintains genomic integrity by unwinding G‐quadruplexes
Source: Genes Cells. 2023 Aug 26;28(10):694–708. doi: 10.1111/gtc.13061 (PMC11447921; doi:10.1111/gtc.13061)
Supplement: Supplementary file 1 — Figure S1. Validation of DHX36 antibodies. (a) Immunofluorescence staining of IMR90 cells transduced with shDHX36 expression vector or control vector using anti‐DHX36 antibodies. Projection of 13 0.75 μm optical sections through the nucleus and cytoplasm encompassing 9 μm. (b) Western blotting analysis using indicated antibodies for cells used in (a). Figure S2. Loss of nuclear BG4 foci after DNase treatment. Fixed IMR90 cells were treated with DNase I or RNase A, or were left untreated. These cells were immunostained with BG4 antibodies. Figure S3. DHX36–RPA complex is retained after DNase I treatment. RPA1‐FLAG, RPA2‐FLAG, or RPA3‐FLAG complex from HEK293FT cells was purified using anti‐FLAG antibody‐attached beads. After subsequent treatment of the beads‐attached complex with DNase I (250 U/μL), proteins were separated on a gel and analyzed using western blotting with DHX36 or FLAG antibody. Figure S4. Reduced survival under PDS treatment in DHX36‐depleted cells. IMR90 cells expressing shDHX36 or control cells harboring shVector were treated with multiple concentrations of PDS for 2 days. Representative images of the cells in the experiment in Figure 6a–c are shown. Figure S5. DHX36 overproduction desensitizes cells to G4 stabilizers. IMR90 cells ectopically expressing DHX36 from viral promoters using a pMXs vector were treated with multiple concentrations of PDS for 2 days. (a) Cell viability was determined by measuring the dehydrogenase activity of living cells after PDS treatment. Values represent mean ± SEM of data from three experiments. (b) Western blotting with the indicated antibodies for whole‐cell extracts. Figure S6. Cells in S‐phase are represented by their higher PCNA signal intensity in the nuclei. (a) Images of IMR90 cells stained with PCNA antibodies and Hoechst 33342 (DNA). Arrowheads indicate nuclei with higher PCNA signal intensity. (b) The signal intensity of PCNA or Hoechst staining per nucleus was calculated using the images in a. Nuclei wer [file GTC-28-694-s001.pdf]

Figure S1

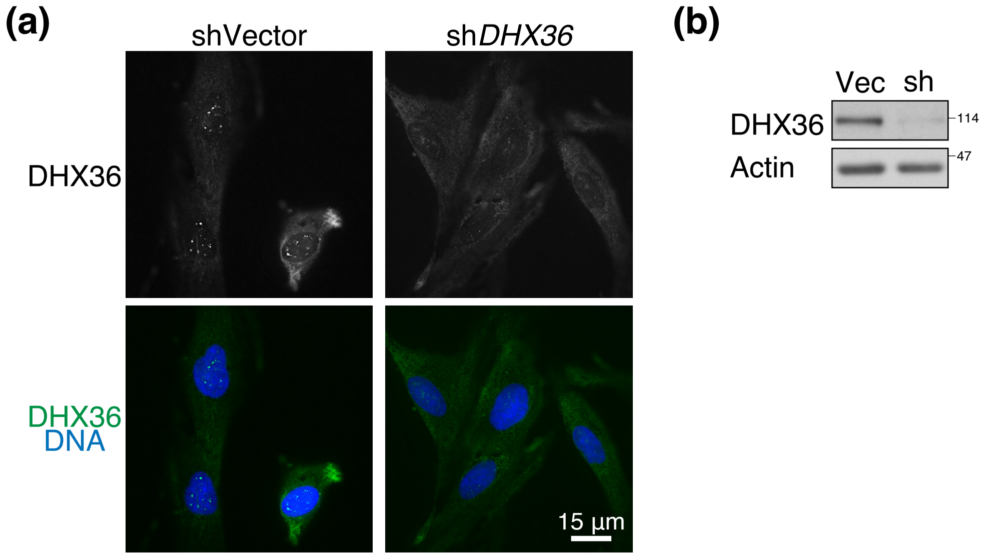

FIGURE S1 Validation of DHX36 antibodies. (a) Immunofluorescence staining of IMR90 cells transduced with sh*DHX36* expression vector or control vector using anti-DHX36 antibodies. Projection of thirteen 0.75 µm optical sections through the nucleus and cytoplasm encompassing 9 µm. (b) Western blotting analysis using indicated antibodies for cells used in (a).

Figure S2

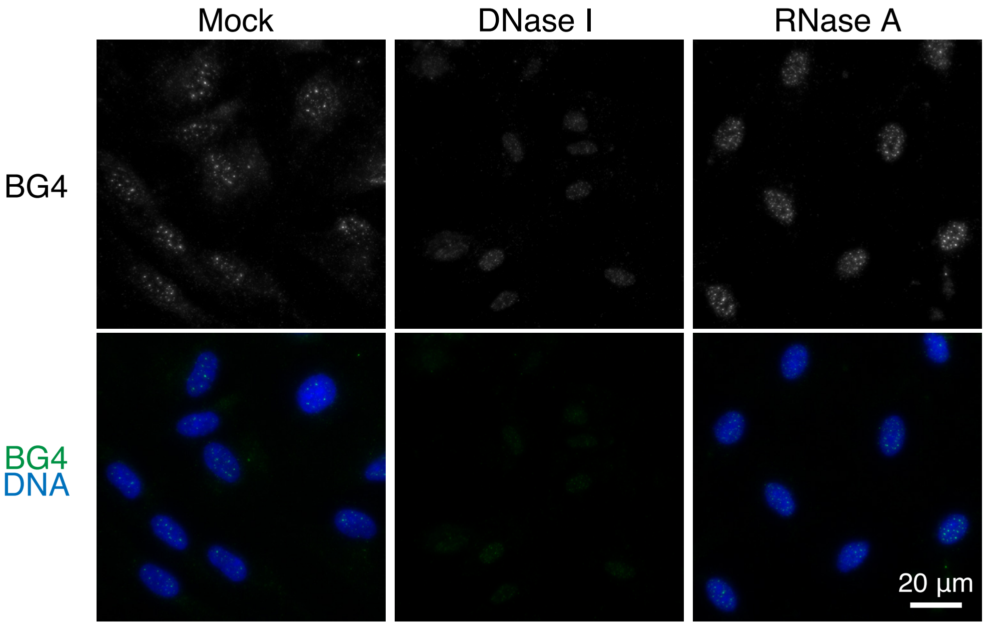

FIGURE S2 Loss of nuclear BG4 foci after DNase treatment. Fixed IMR90 cells were treated with DNase I or RNase A, or were left untreated. These cells were immunostained with BG4 antibodies.

Figure S3

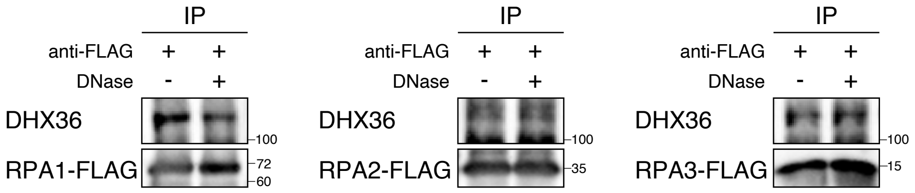

FIGURE S3 DHX36–RPA complex is retained after DNase I treatment. RPA1-FLAG, RPA2-FLAG, or RPA3-FLAG complex from HEK293FT cells was purified using anti-FLAG antibody-attached beads. After subsequent treatment of the beads-attached complex with DNase I (250 U/μL), proteins were separated on a gel and analyzed using western blotting with DHX36 or FLAG antibody.

Figure S4

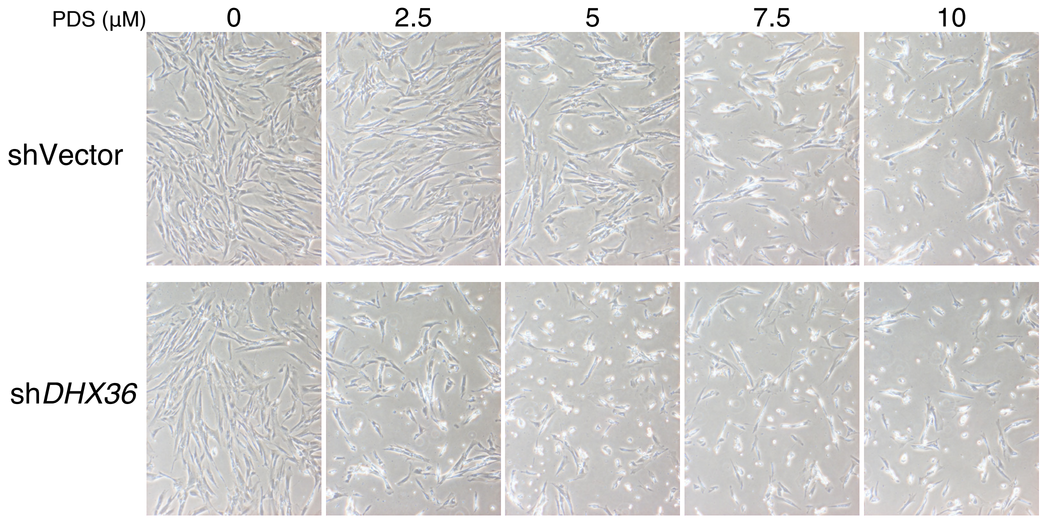

FIGURE S4 Reduced survival under PDS treatment in DHX36-depleted cells. IMR90 cells expressing sh*DHX36* or control cells harboring shVector were treated with multiple concentrations of PDS for two days. Representative images of the cells in the experiment in Figure 6a, b, c are shown.

Figure S5

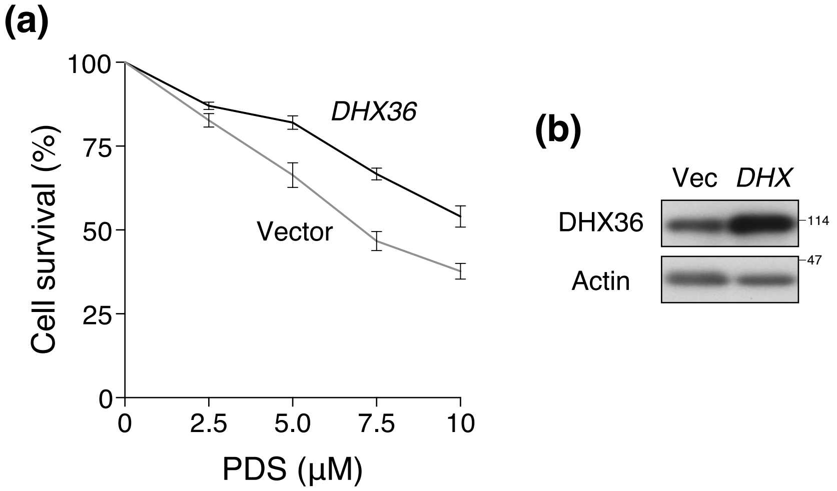

FIGURE S5 DHX36 overproduction desensitizes cells to G4 stabilizers. IMR90 cells ectopically expressing DHX36 from viral promoters using a pMXs vector were treated with multiple concentrations of PDS for two days. (a) Cell viability was determined by measuring the dehydrogenase activity of living cells after PDS treatment. Values represent mean ± SEM of data from three experiments. (b) Western blotting with the indicated antibodies for whole-cell extracts.

Figure S6

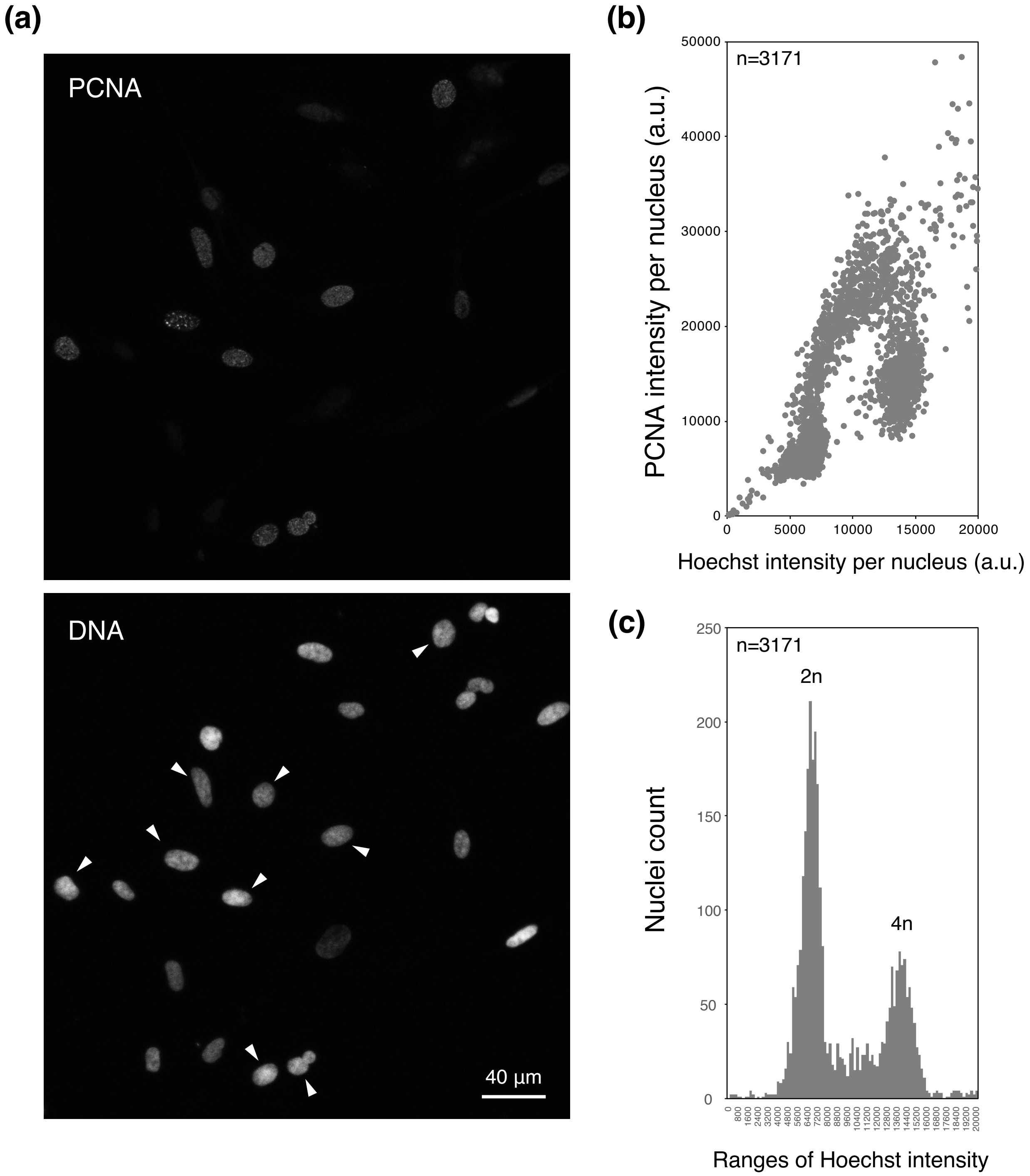

FIGURE S6 Cells in S-phase are represented by their higher PCNA signal intensity in the nuclei. (a) Images of IMR90 cells stained with PCNA antibodies and Hoechst 33342 (DNA). Arrowheads indicate nuclei with higher PCNA signal intensity. (b) The signal intensity of PCNA or Hoechst staining per nucleus was calculated using the images in a. Nuclei were plotted according to PCNA and Hoechst signal intensities (n=3171). (c) Cell cycle profiles of cells in b. Populations with DNA content over 4n are likely to be clumping cells on a coverslip (b and c).

Figure S7

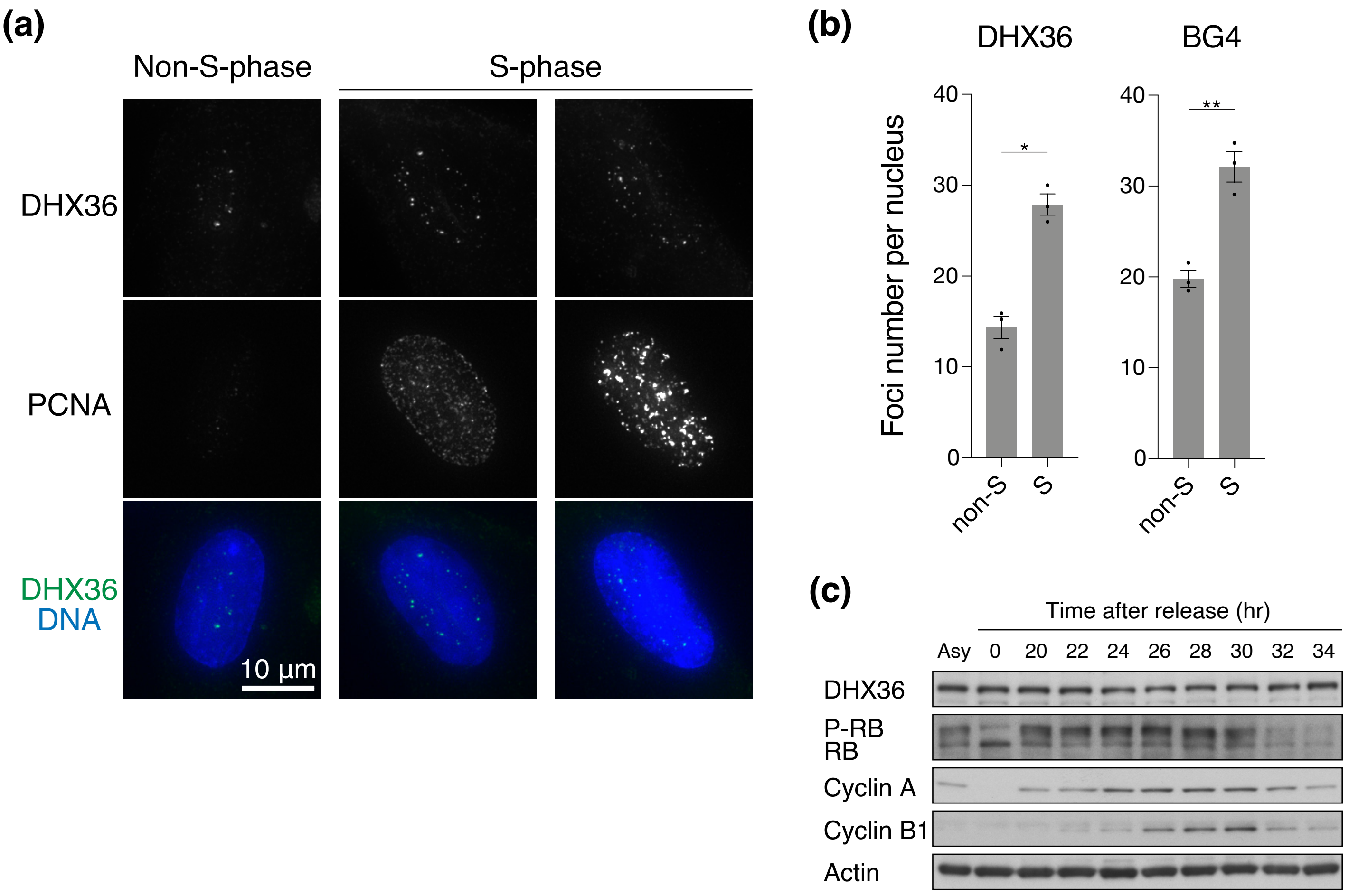

FIGURE S7 Nuclear DHX36 and BG4 signals increase in the S-phase. (a) Representative images of nuclei stained with DHX36 and PCNA antibodies. S-phase nuclei were identified by their higher PCNA intensities. (b) Average of nuclear foci of G4 or DHX36 in the S-phase or non-S-phase. At least 30 nuclei were counted for each stage. (c) DHX36 protein levels remained unchanged throughout the cell cycle, as seen using western blotting with the indicated antibodies for whole-cell extracts. Cells were synchronized in the G0 phase by incubating with low-serum media for 2–3 days and released into the cell cycle for the period shown above. Values are represented as mean ± SEM of data from three experiments. Statistical significance was calculated using two-tailed paired *t*-tests. $^{**}p < 0.01$, $^{*}p < 0.05$. Asy, asynchronous cells.

Figure S8

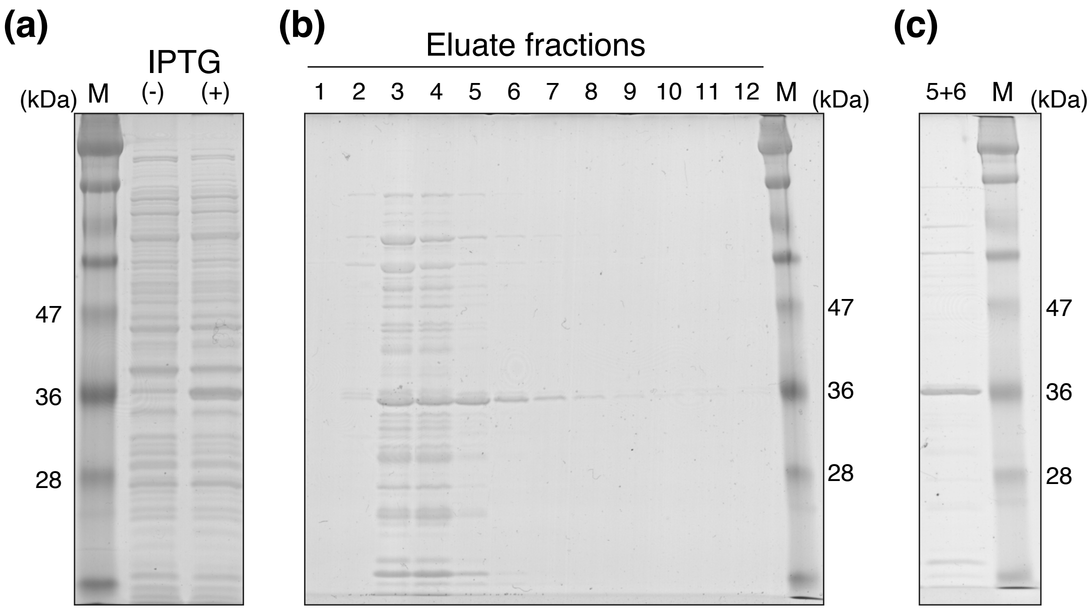

FIGURE S8 Expression and purification of BG4 scFv. (a) Whole-cell extracts of *Escherichia coli* cells harboring pSANG10-3F-BG4 with or without IPTG-induced expression of 6×His-tagged BG4 single-chain variable fragment (scFv). (b) His-tagged BG4 scFv was collected using a metal affinity resin and eluted in a buffer containing imidazole. (c) Eluate fractions No. 5 and 6 from b were combined and dialyzed against PBS buffer. This purified BG4 scFv was used for immunofluorescence. All the gels were stained with Coomassie Brilliant Blue.
